# Differentiation of pathological subtypes and Ki-67 and TTF-1 expression by dual-energy CT (DECT) volumetric quantitative analysis in non-small cell lung cancer

**DOI:** 10.1186/s40644-024-00793-6

**Published:** 2024-10-25

**Authors:** Yuting Wu, Jingxu Li, Li Ding, Jianbin Huang, Mingwang Chen, Xiaomei Li, Xiang Qin, Lisheng Huang, Zhao Chen, Yikai Xu, Chenggong Yan

**Affiliations:** 1 Department of Medical Imaging Center, Nanfang Hospital, Southern Medical University, Guangzhou, 510515 China; 2 Department of Radiology, The First Affiliated Hospital of Guangzhou Medical University, Guangzhou, 510120 China

**Keywords:** Dual-energy CT, Non-small cell lung cancer, Thyroid transcription factor 1, Quantitative analysis

## Abstract

**Background:**

To explore the value of dual-energy computed tomography (DECT) in differentiating pathological subtypes and the expression of immunohistochemical markers Ki-67 and thyroid transcription factor 1 (TTF-1) in patients with non-small cell lung cancer (NSCLC).

**Methods:**

Between July 2022 and May 2024, patients suspected of lung cancer who underwent two-phase contrast-enhanced DECT were prospectively recruited. Whole-tumor volumetric and conventional spectral analysis were utilized to measure DECT parameters in the arterial and venous phase. The DECT parameters model, clinical-CT radiological features model, and combined prediction model were developed to discriminate pathological subtypes and predict Ki-67 or TTF-1 expression. Multivariate logistic regression analysis was used to identify independent predictors. The diagnostic efficacy was assessed by the area under the receiver operating characteristic curve (AUC) and compared using DeLong’s test.

**Results:**

This study included 119 patients (92 males and 27 females; mean age, 63.0 ± 9.4 years) who was diagnosed with NSCLC. When applying the DECT parameters model to differentiate between adenocarcinoma and squamous cell carcinoma, ROC curve analysis indicated superior diagnostic performance for conventional spectral analysis over volumetric spectral analysis (AUC, 0.801 vs. 0.709). Volumetric spectral analysis exhibited higher diagnostic efficacy in predicting immunohistochemical markers compared to conventional spectral analysis (both *P* < 0.05). For Ki-67 and TTF-1 expression, the combined prediction model demonstrated optimal diagnostic performance with AUC of 0.943 and 0.967, respectively.

**Conclusions:**

The combined predictive model based on volumetric quantitative analysis in DECT offers valuable information to discriminate immunohistochemical expression status, facilitating clinical decision-making for patients with NSCLC.

**Supplementary Information:**

The online version contains supplementary material available at 10.1186/s40644-024-00793-6.

## Background

According to the latest global burden of cancer reported by the International Agency for Research on Cancer, lung cancer has the second highest incidence rate and is the leading cause of cancer-related mortality worldwide [1]. Among them, non-small cell lung cancer (NSCLC) constitutes approximately of 85% of lung cancer cases and is the most prevalent pathological subtype [2, 3]. The histologic classification and molecular phenotype of NSCLC plays a crucial role in its diagnosis and personalized treatment [4, 5].

The molecular phenotype of a tumor plays a crucial role in its diagnosis and personalized treatment [6]. Among these, immunohistochemical markers, such as cellular proliferation biomarker Ki-67, highly correlates with clinical decision-making and prognostic assessment in NSCLC [7, 8]. Ki-67 is a marker associated with cellular proliferation and has been identified as a poor prognostic factor for survival in NSCLC [9]. High Ki-67 levels may suggest tumor biological behavior and response to chemotherapy [10]. Recent studies have shown that thyroid transcription factor 1 (TTF-1) is an important nuclear transcription factor expressed in NSCLC, playing a pivotal role in regulating lung carcinogenesis, progression, and prognosis [11, 12]. TTF-1 is frequently expressed in lung cancer and serves as an important marker to distinguish primary lung adenocarcinomas from other metastatic tumors [13]. TTF-1 is also associated with tumor differentiation and survival outcomes in lung cancer [14]. Consequently, the identification of immunohistochemical biomarkers plays a critical role in the understanding of the biological mechanisms underlying lung cancer, guiding clinical decision-making and treatment planning.

Histopathology and immunohistochemistry serve as the gold standard for diagnosing the pathological type and biomarker expression in NSCLC. However, obtaining tissue samples necessitates invasive procedures like percutaneous puncture biopsy or bronchoscopy, posing risks of bleeding, pneumothorax, and potential tumor enlargement [15, 16]. Coupled with the fact that traditional stained slide preparation is time-consuming, labor-intensive and error-prone [17]. Consequently, exploring non-invasive imaging modalities holds great significance for providing an effective adjunctive diagnosis for patients with NSCLC.

With rapid advances in medical imaging technology, the emergence of dual-energy CT (DECT) is capable of generating virtual monoenergetic and material decomposition images based on spectral imaging data [18, 19]. Currently, quantitative analysis parameters derived from DECT, which reflects the functional and biological characteristics of the lesions, have potential applications in differentiating pathological subtypes and assessing treatment response of lung cancer [20–23]. For example, a study by Li et al. [23] demonstrated that the iodine concentration (IC) derived from DECT imaging, particularly in the venous phase, serves as a valuable indicator for assessing tumor angiogenesis and prognosis. Recently, extracellular volume fraction (ECV) has been introduced to reflect changes in the cellular microenvironment, as determined from contrast-enhanced DECT images [24–27]. However, most previous studies have relied on conventional two-dimensional region-based spectral analysis methods on the largest tumor area, which fail to fully represent the comprehensive information of the whole lesion. Furthermore, only a few studies have explored the potential synergy between subjective radiological features and quantitative DECT parameters in developing prediction model in NSCLC.

This study aims to investigate the value of quantitative DECT volumetric spectral analysis for distinguishing the pathological subtypes and immunohistochemical expressions of Ki-67 and TTF-1 in NSCLC through a bi-center clinical study. Furthermore, we integrated a DECT parameters with clinical and CT radiological features to build a combined predictive model, aimed at tailoring a personalized treatment strategy for these patients.

## Materials and methods

### Patients

This study was approved by our institutional review board, and all participants provided informed consent. This study was conducted in accordance with the Declaration of Helsinki (revised 2013). Between July 2022 and May 2024, patients who underwent contrast enhanced chest DECT for suspected lung cancer were prospectively enrolled at the Nanfang Hospital of Southern Medical University (Center-1) or the First Affiliated Hospital of Guangzhou Medical University (Center-2).

Inclusion criteria were as follows: (I) patients presented as solid nodule or mass on CT images; (II) DECT within one month before surgery; (III) no prior clinical anti-tumor therapy before enrollment. The exclusion criteria were as follows: (I) incomplete clinical information or without Ki-67 and TTF-1 immunohistochemistry (*n* = 22); (II) inadequate image quality or inability to completely outline the lesion (*n* = 31); (III) histopathological diagnosis of benign lesions or other types of lung cancer including small-cell lung cancer by biopsy or surgical resection (*n* = 75); and (IV) tumor size less than 10 mm (*n* = 7).

**Fig. 1:**
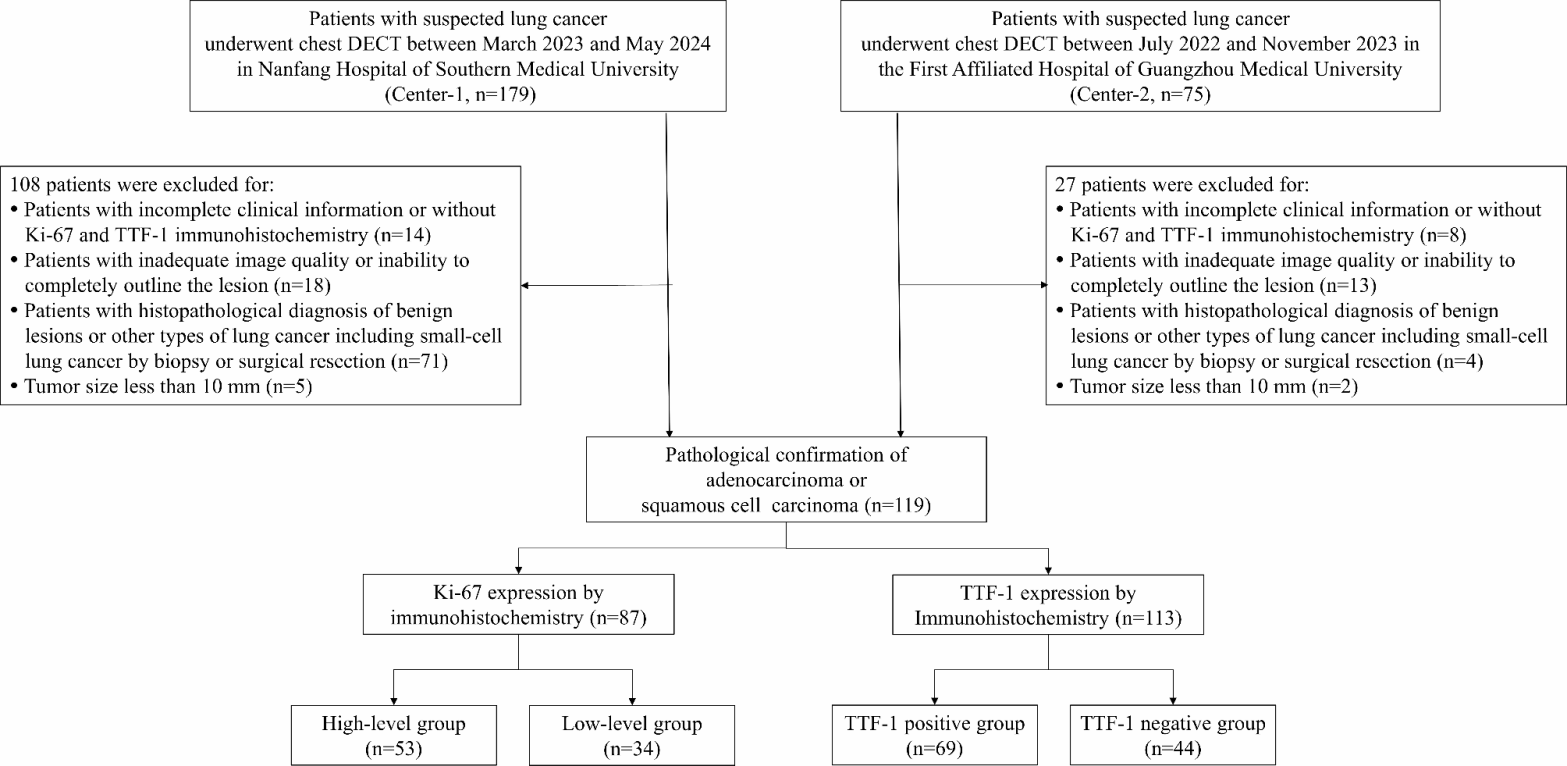
Flowchart of study population. Numbers in parentheses are number of patients. DECT, dual-energy computed tomography

Finally, 119 patients with pathologically confirmed NSCLC were enrolled. Clinical information, including age, gender, histological type, smoking history, alcohol consumption history, and family tumor history, was collected. A flowchart of the study population is shown in Fig. 1.

### CT imaging protocols

Chest CT examinations were performed on a DECT scanner [Revolution (Center-1) or Apex (Center-2) CT; GE Healthcare Chicago, IL, USA] in gemstone spectral imaging mode, scanning range from lung apex to diaphragm. Detailed scanning parameters were as follows: tube voltages of 80/140 kV fast switching, tube current of 320–370 mA, tube rotation time of 0.5 s, and automatic reconstruction of images with 1.25 mm slice thickness at 1.25-mm interval. A total of 60–70 mL of non-ionic iodinated contrast agent (Ioprimor Injection, 350–370 mg/L, Bayer Schering Pharma, Berlin, Germany) was injected through the antecubital vein at a rate of 2.0–3.0 mL/s. The dual-phase contrast enhanced CT for the arterial phase (AP) and venous phase (VP) scan was performed 30 and 60 s after the start of injection, respectively.

### Image analysis

#### Evaluation of CT radiological features

CT characteristics were subjectively assessed with the lung [window width, 1,500 Hounsfield units (HU); window level − 600 HU] and mediastinal (window width, 350 HU; window level, 45 HU) window settings. The radiological features (tumor size, lobulation, spiculation, air bronchogram, pleural retraction, obstructive change, vascular invasion, rim enhancement, necrosis, and lymph node enlargement) were documented for each patient by one radiologist (Y.W.) with 3 years of experience, and confirmed by another senior radiologist (C.Y.) with 13 years of experience. Both radiologists were blinded to patient information and pathological findings. A consensus was reached through discussion if disagreement occurred. Detailed information on clinical and subjective radiological characteristics is provided in Table S1.

#### DECT parameters measurement and quantitative analysis

All images were transferred to a post-processing workstation (GE AW 4.7), which were measured independently by two experienced radiologists (Y.W. and J.L.) using the volume rendering and GSI Viewer software on a mediastinal window (window width: 350 HU, window level: 45 HU). If multiple lesions are present, the lesion with the largest diameter was chosen for analysis. The parameters measured by the two radiologists were averaged for analysis and comparison.

Two different quantitative parameters measurement methods were performed: (I) Volumetric spectral analysis: the volume of interest (VOI) was delineated around the tumor contour in a semi-automated manner with a combination of manual correction, avoiding obvious adjacent and bronchi. (II) Conventional spectral analysis: elliptical regions of interest (ROIs) were drawn to cover the largest possible area of the lesion in three consecutive slices, avoiding necrosis, vascularity, and calcification, and the mean values were then calculated for analysis. The size, shape, and position of the VOIs or ROIs were kept consistent using the copy-and-paste function between the unenhanced and enhanced data. Subsequently, a series of parameters including monochromatic CT numbers at energy levels of 40–140 keV, effective atomic number (Zeff), iodine concentration (IC), and water concentration (WC) were measured for the entire lesion.

To minimize the effect on individual circulatory status and scan time, the lesion IC value was normalized to the aorta IC value at the same level to calculate the normalized iodine concentration (NIC): NIC = IC_**lesion**_/IC_**aorta**_. The slopes of the energy attenuation curves were calculated, including K_40 − 70_ calculated from (CT_40keV_ - CT_70keV_)/30 and K_40 − 100_ calculated from (CT_40keV_ – CT_100keV_)/60. According to the ECV formula: ECV (%) = (1 - hematocrit) × (IC_**lesion**_/IC_**aorta**_) ×100%, where IC_**lesion**_ and IC_**aorta**_ were the lesion and aortic iodine concentration at the venous phase, respectively. Arterial enhancement fraction (AEF) = (IC_AP_/IC_VP_) ×100% and normalized arterial enhancement fraction (NAEF) = (NIC_AP_/NIC_VP_) ×100% were calculated for the lesion.

#### Immunohistochemistry staining of Ki-67 and TTF-1

The pathological type and immunohistochemical staining results of the tumor specimens were recorded. The Ki-67 expression level was divided into low expression group (≤ 30%) and high expression group (> 30%). The number of positive cells < 10% with light staining was defined as TTF-1 negative, while the number of positive cells ≥ 10% with brownish-yellow granules was defined as TTF-1 positive.

### Statistical analysis

Statistical analyses were performed using SPSS (version 26.0, IBM, USA) and GraphPad Prism (version 9.5.0, GraphPad Software, USA). A *P* value < 0.05 was considered statistically significant. Normality of variance was assessed by the one-sample Kolmogorov-Smirnov (K-S) test. Categorical variables were tested using the chi-squared test or Fisher’s exact probability method. Comparisons were made using the Student t-test for normally distributed continuous variables or the Mann-Whitney test for non-normally distributed continuous variables.

Interobserver agreement for the measurement of spectral parameters was assessed using intraclass correlation coefficients (ICCs) and kappa (κ) index for radiological features. The degree of agreement was interpreted as follows: an ICC or κ value of 0.00-0.20, poor agreement; 0.21–0.40, fair agreement; 0.41–0.60, moderate agreement; 0.61–0.80, substantial agreement; and 0.81-1.00, almost perfect agreement.

Significant variables from the univariate analyses were included in multivariate logistic regression analyses to derive independent predictors, and odds ratios (ORs) were calculated. Additionally, receiver operating characteristic (ROC) curve analyses were performed to assess the diagnostic performance of the model by area under the ROC curve (AUCs) and compared by the DeLong’s test. The Hosmer-Lemeshow goodness of fit test was used to test the fit of the model. Youden’s index (sensitivity plus specificity) was calculated to determine the optimal cut-off value for the prediction model.

## Results

### Patient characteristics

A total of 119 patients (92 males and 27 females; mean age, 63.0 ± 9.4 years) who had NSCLC with DECT images were included. Following pathological confirmation, the cohort comprised 84 cases of adenocarcinoma and 35 cases of squamous carcinoma. The clinical characteristics are summarized in Table 1.

**Table 1:** Demographic and clinical characteristics of patients

Characteristics	Result
Patient number	119
Gender	
Male	92(77.3)
Female	27(22.7)
Age(years)	63.0 ± 9.4
Histological type	
Adenocarcinoma	84(70.6)
Squamous cell carcinoma	35(29.4)
Tumor Location	
Central	49(41.2)
Peripheral	70(58.8)
Lobe location	
Right upper	34(28.6)
Right middle	13(10.9)
Right lower	28(23.5)
Left upper	30(25.2)
Left lower	14(11.8)
Smoking history	
Yes	71(59.7)
No	48(40.3)
Drinking history	
Yes	27(22.7)
No	92(77.3)
Family tumor history	
Yes	7(5.9)
No	112(94.1)
Serum tumor markers	
NSE (ng/ml)	
High (+)	46(38.7)
Normal (-)	73(61.3)
CYFRA21 − 1 (ng/ml)	
High (+)	77(64.7)
Normal (-)	42(35.3)
CEA (ng/mL)	
High (+)	69(58.0)
Normal (-)	50(42.0)
SCCA (ng/ml)	
High (+)	32(26.9)
Normal (-)	87(73.1)
Clinical staging	
I+II	17(14.3)
III+IV	102(85.7)

Data are numbers of patients, with percentage in parentheses. NSE, neuron-specific enolase; CYFRA21 − 1, cytokeratin 19 fragment; CEA, carcinoembryonic antigen; SCCA, squamous cell carcinoma antigen

### Interobserver reproducibility

The interobserver agreement of DECT parameter measurements in volumetric and conventional spectral analysis is shown in Tables S2 and S3, respectively. For volumetric spectral analysis, the agreement for DECT parameters was substantial (ICCs = 0.879–0.989). For conventional spectral analysis, the agreement ranged from moderate to substantial (ICCs = 0.785–0.992) for DECT parameters. Interobserver agreement for subjective CT radiological features ranged from substantial to almost perfect (κ = 0.750-1.000), as shown in Table S4.

### Differential diagnosis efficiency of the two spectral CT analysis methods for ADC and SQCC

**Fig. 2:**
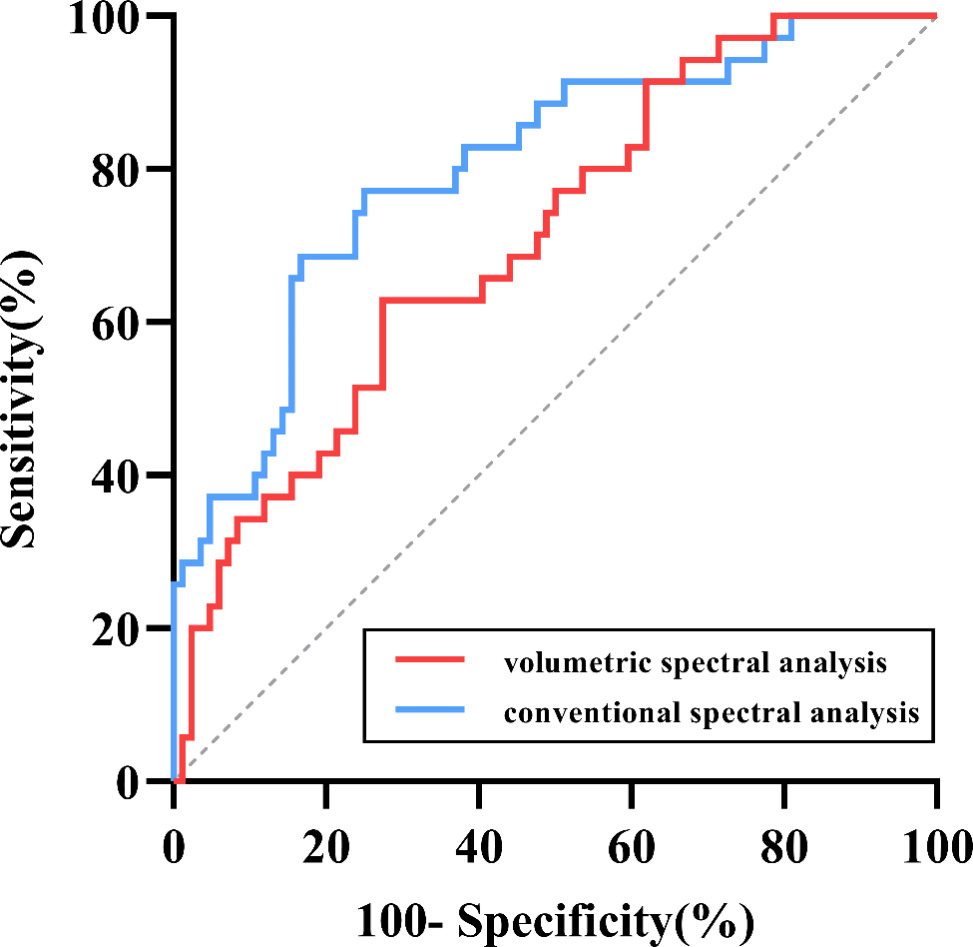
ROC curves for the differential diagnosis of adenocarcinoma and squamous cell carcinoma by the volumetric and conventional spectral analyses

The volumetric and conventional spectral analysis results are summarized in Table 2 (See Supplementary Material for more details). The diagnostic efficiency of the significant features and the combined model in discriminating ADC from SQCC is shown in Table S5. Among the volumetric spectral analysis results, the diagnostic efficacy of CT_140keV_ in AP and VP (AUC of 0.703) was the highest. The diagnostic efficacy was improved by the DECT parameters integrated model (AUC of 0.709, 72.6% sensitivity, 62.9% specificity, and 92.9% accuracy). In conventional spectral analysis, the diagnostic efficacy of AEF and NIC in AP was the highest (AUC of 0.677). As shown in Fig. 2, the conventional DECT parameters integrated model outperformed volumetric spectral analysis, achieving an AUC of 0.801, 75.0% sensitivity, 77.1% specificity, 85.1% accuracy, and 91.7% accuracy.

**Table 2:** Comparison of the spectral CT analysis results for lung cancer

parameter	Volumetric spectral analysis			Conventional spectral analysis		
	ADC (*n* = 84)	SQCC (*n* = 35)	*P* value	ADC (*n* = 84)	SQCC (*n* = 35)	*P* value
**Arterial Phase**						
CT40-keV (HU)	135.14 ± 35.58	121.97 ± 29.73	0.056	134.43 ± 33.69	120.25 ± 33.52	0.058
CT60-keV (HU)	71.15 ± 15.07	68.61 ± 12.50	0.382	71.73 ± 14.05	68.56 ± 14.64	0.270
CT70-keV (HU)	55.99 ± 10.98	55.98 ± 8.86	0.998	56.89 ± 10.34	56.36 ± 10.77	0.801
CT80-keV (HU)	46.30 ± 9.02	47.91 ± 6.94	0.347	47.41 ± 8.76	48.53 ± 8.79	0.526
CT100-keV (HU)	35.42 ± 7.95	38.83 ± 5.63	**0.009**	36.74 ± 8.19	39.73 ± 7.47	0.065
CT120-keV (HU)	30.16 ± 8.03	34.47 ± 5.55	**0.001**	31.60 ± 8.50	35.52 ± 7.33	**0.019**
CT140-keV (HU)	27.00 ± 8.25	31.81 ± 5.66	**<0.001**	28.59 ± 8.50	32.93 ± 7.42	**0.010**
K40–70	2.64 ± 0.90	2.20 ± 0.74	**0.012**	2.58 ± 0.88	2.13 ± 0.83	**0.010**
K40–100	1.66 ± 0.57	1.39 ± 0.47	**0.012**	1.63 ± 0.55	1.34 ± 0.52	**0.010**
Zeff	8.43 ± 0.27	8.29 ± 0.23	**0.010**	8.43 ± 0.26	8.29 ± 0.25	**0.008**
IC (mg/cm^3^)	13.69 ± 4.82	11.36 ± 3.95	**0.013**	13.74 ± 4.66	11.31 ± 4.37	**0.009**
NIC	0.15 ± 0.06	0.12 ± 0.05	**0.009** ^*^	0.15 ± 0.06	0.12 ± 0.06	**0.002** ^*^
WC (mg/cm^3^)	1018.95 ± 9.37	1024.31 ± 6.27	**0.001** ^*^	1021.18 ± 10.02	1026.30 ± 7.56	**0.004** ^*^
**Venous Phase**						
CT40-keV (HU)	159.03 ± 37.94	156.82 ± 33.12	0.764	159.43 ± 36.49	153.15 ± 38.07	0.400
CT60-keV (HU)	82.52 ± 16.75	84.72 ± 14.30	0.499	83.96 ± 16.60	83.79 ± 16.04	0.959
CT70-keV (HU)	64.52 ± 12.65	67.74 ± 10.04	0.183	66.05 ± 12.63	67.09 ± 11.47	0.675
CT80-keV (HU)	53.00 ± 10.49	56.85 ± 7.88	0.053	54.69 ± 10.52	56.47 ± 8.99	0.382
CT100-keV (HU)	40.08 ± 8.88	44.61 ± 6.34	**0.002**	41.80 ± 9.17	44.50 ± 7.09	0.123
CT120-keV (HU)	33.82 ± 8.55	38.70 ± 6.17	**0.001** ^*^	35.59 ± 8.94	38.72 ± 6.77	**0.041**
CT140-keV (HU)	29.95 ± 8.70	34.91 ± 6.86	**0.001** ^*^	31.89 ± 8.97	35.25 ± 6.80	**0.049**
K40–70	3.15 ± 0.92	2.97 ± 0.82	0.315	3.11 ± 0.88	2.87 ± 0.95	0.181
K40–100	1.98 ± 0.57	1.87 ± 0.52	0.319	1.96 ± 0.56	1.81 ± 0.59	0.191
Zeff	8.57 ± 0.26	8.52 ± 0.24	0.314	8.59 ± 0.26	8.52 ± 0.27	0.216
IC (mg/cm^3^)	16.33 ± 4.68	15.40 ± 4.35	0.316	16.55 ± 4.69	15.30 ± 4.95	0.196
NIC	0.42 ± 0.13	0.36 ± 0.08	**0.006**	0.43 ± 0.13	0.36 ± 0.09	**0.009**
WC (mg/cm^3^)	1020.74 ± 9.04	1025.40 ± 6.92	**0.002** ^*^	1023.29 ± 9.40	1026.47 ± 7.11	**0.047**
ECV	0.25 ± 0.08	0.23 ± 0.06	**0.047** ^*^	0.26 ± 0.08	0.23 ± 0.06	**0.036**
AEF	0.85 ± 0.18	0.75 ± 0.21	**0.010**	0.84 ± 0.17	0.75 ± 0.22	**0.002** ^*^
NAEF	0.36 ± 0.10	0.34 ± 0.12	0.069^*^	0.36 ± 0.10	0.34 ± 0.12	0.077^*^

Data are presented as mean ± standard deviation; *, Mann-Whitney test; K_40–70_ = (CT_40 − keV_- CT_70 − keV_)/30; K_40–100_ = (CT_40 − keV_- CT_100 − keV_)/60; Zeff, effective atomic number; (N)IC, (normalized) iodine concentration; WC, water concentration; ECV(%)=(1-hematocrit)×NIC; (N)AEF, (normalized) arterial enhancement fraction

### Multivariate logistic analysis and prediction of Ki-67 expression model performance

The characteristics of DECT parameters and significant radiological features between groups with different levels of Ki-67 expression are summarized in Table 3 and S6 (See Supplementary Material for more details). As shown in Table S7, the results showed that Zeff-AP (OR = e^43.710^, 95% CI: 3.473- e^86.175^, *P* = 0.044), Zeff-VP (OR = 1/e^180.668^, 95% CI: 1/e^339.530^-1/e^221.806^, *P* = 0.026), IC-VP (OR = e^9.424^, 95% CI: 2.345- e^17.995^, *P* = 0.031), ECV-VP (OR = e^52.531^, 95% CI: 31.784- e^101.604^, *P* = 0. 036), gender (OR = 0.123, 95% CI: 0.018–0.820, *P* = 0.030), pleural retraction (OR = 0.079, 95%CI: 0.014–0.433, *P* = 0.003) and lymph node enlargement (OR = 11.626, 95% CI: 1.150-117.538, *P* = 0.038) were independent factors of the Ki-67 expression prediction model. The detailed equations were as follows: logit (P) = 43.710 × Zeff-AP + (-180.668) × Zeff-VP + 9.424 × IC-VP + 52.531 × ECV-VP + (-2.099) × gender (male = 1; female = 2) + (-2.540) × pleural retraction (presence = 1; absence = 0) + 2.453× lymph node enlargement (presence = 1; absence = 0)-1725.321, where ‘P’ is the probability of Ki-67 expression level. (See Supplementary Material for the calculation formula of ‘P’).

**Table 3:** Comparison of the spectral CT analysis results for Ki-67 expression

parameter	Volumetric spectral analysis			Conventional spectral analysis		
	Low-level (*n* = 34)	High-level (*n* = 53)	*P* value	Low-level (*n* = 34)	High-level (*n* = 53)	*P* value
**Arterial Phase**						
CT40-keV (HU)	146.72 ± 35.28	129.42 ± 31.17	0.050^*^	144.65 ± 29.95	127.37 ± 33.22	**0.013** ^*^
CT60-keV (HU)	75.05 ± 13.88	70.35 ± 13.61	0.123	75.32 ± 10.46	70.27 ± 14.75	0.086
CT70-keV (HU)	58.04 ± 9.76	56.36 ± 10.17	0.447	58.91 ± 7.43	56.74 ± 11.14	0.279
CT80-keV (HU)	47.21 ± 7.95	47.42 ± 8.54	0.907	48.46 ± 6.96	48.09 ± 9.42	0.837
CT100-keV (HU)	35.00 ± 7.46	37.38 ± 7.64	0.157	36.66 ± 8.08	38.38 ± 8.39	0.348
CT120-keV (HU)	29.11 ± 7.93	32.54 ± 7.67	**0.048**	30.96 ± 9.14	33.69 ± 8.37	0.155
CT140-keV (HU)	25.58 ± 8.37	29.61 ± 7.82	**0.025**	27.77 ± 9.12	30.86 ± 8.49	0.112
K40–70	2.96 ± 0.94	2.44 ± 0.78	**0.006**	2.86 ± 0.88	2.35 ± 0.83	**0.008** ^*^
K40–100	1.86 ± 0.59	1.53 ± 0.49	**0.006**	1.80 ± 0.55	1.48 ± 0.52	**0.008** ^*^
Zeff	8.52 ± 0.28	8.36 ± 0.24	**0.006**	8.52 ± 0.26	8.36 ± 0.25	**0.008** ^*^
IC (mg/cm^3^)	15.40 ± 5.01	12.62 ± 4.14	**0.006**	15.19 ± 4.67	12.52 ± 4.38	**0.008** ^*^
NIC	0.17 ± 0.06	0.14 ± 0.06	0.080^*^	0.16 ± 0.06	0.14 ± 0.06	**0.045** ^*^
WC (mg/cm^3^)	1016.44 ± 9.95	1021.81 ± 8.37	**0.005** ^*^	1019.36 ± 11.54	1023.85 ± 8.94	**0.039** ^*^
**Venous Phase**						
CT40-keV (HU)	168.36 ± 32.59	147.66 ± 34.03	**0.006**	164.98 ± 26.61	148.12 ± 34.45	**0.017**
CT60-keV (HU)	85.34 ± 13.41	78.99 ± 15.56	0.053	85.59 ± 10.97	80.02 ± 15.72	0.075
CT70-keV (HU)	65.99 ± 10.50	62.78 ± 11.60	0.196	66.75 ± 8.33	63.87 ± 12.02	0.191
CT80-keV (HU)	53.60 ± 9.20	52.39 ± 9.57	0.562	54.83 ± 7.11	53.59 ± 10.16	0.504
CT100-keV (HU)	39.71 ± 8.58	40.74 ± 8.03	0.573	41.21 ± 7.38	42.01 ± 8.88	0.661
CT120-keV (HU)	32.96 ± 8.66	35.11 ± 7.73	0.231	34.67 ± 7.78	36.42 ± 8.71	0.344
CT140-keV (HU)	28.60 ± 9.19	31.74 ± 7.70	0.089	30.79 ± 8.11	33.06 ± 8.75	0.228
K40–70	3.41 ± 0.84	2.83 ± 0.82	**0.002**	3.27 ± 0.72	2.81 ± 0.84	**0.009**
K40–100	2.14 ± 0.51	1.78 ± 0.51	**0.002**	2.06 ± 0.45	1.77 ± 0.53	**0.009**
Zeff	8.65 ± 0.22	8.48 ± 0.24	**0.002**	8.64 ± 0.21	8.50 ± 0.25	**0.007**
IC (mg/cm^3^)	17.57 ± 3.93	14.72 ± 4.34	**0.003**	17.41 ± 3.79	14.94 ± 4.44	**0.009**
NIC	0.45 ± 0.13	0.37 ± 0.11	**0.005**	0.44 ± 0.12	0.38 ± 0.12	**0.026**
WC (mg/cm^3^)	1018.86 ± 9.49	1022.79 ± 7.93	0.052^*^	1021.78 ± 9.15	1025.03 ± 8.81	0.108^*^
ECV	0.27 ± 0.07	0.23 ± 0.07	**0.006**	0.27 ± 0.07	0.23 ± 0.08	**0.034**
AEF	0.87 ± 0.14	0.87 ± 0.17	0.934	0.87 ± 0.14	0.84 ± 0.19	0.454
NAEF	0.37 ± 0.08	0.39 ± 0.11	0.434^*^	0.37 ± 0.08	0.38 ± 0.13	0.639^*^

Data are presented as mean ± standard deviation; *, Mann-Whitney test; K_40**–**70_ = (CT_40 − keV_- CT_70 − keV_)/30; K_40**–**100_ = (CT_40 − keV_- CT_100 − keV_)/60; Zeff, effective atomic number; (N)IC, (normalized) iodine concentration; WC, water concentration; ECV(%)=(1-hematocrit)×NIC; (N)AEF, (normalized) arterial enhancement fraction

**Fig. 3:**
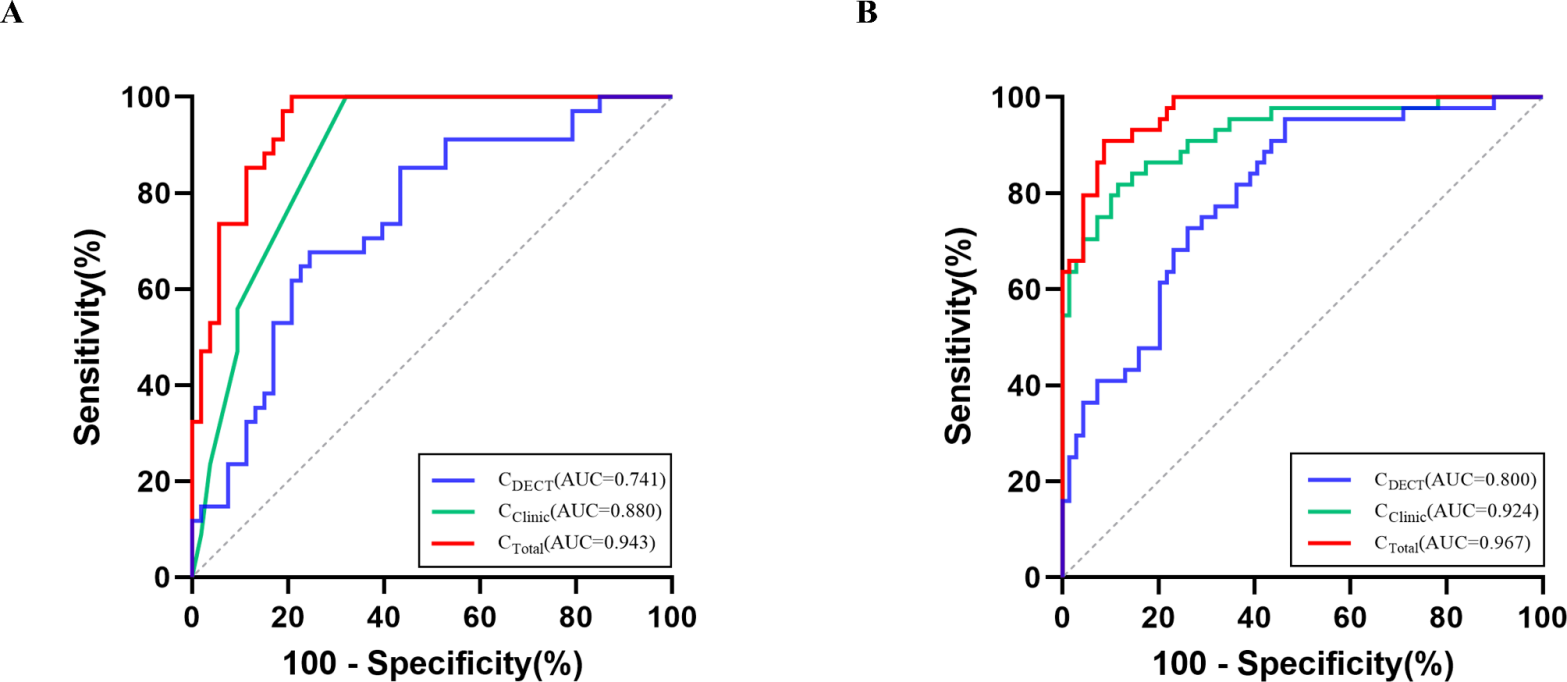
ROC curves for the diagnostic performance of different models for prediction of Ki-67 **(A)** and TTF-1 **(B)** expression. C_DECT_, DECT parameters model; C_Clinic_, Clinical-CT radiological features model; C_Total_, Combined predictive model

The Hosmer-Lemeshow goodness-of-fit test yielded a non-significant result (*P* = 0.558), suggesting a satisfactory fit of the model. The combined predictive model integrating all seven independent factors showed a high diagnostic efficacy with an AUC of 0.943, sensitivity of 79.2%, specificity of 100.0%, and accuracy of 85.1%. As shown in Fig. 3A, the diagnostic performance of the combined predictive model (AUC = 0.943, *P* < 0.001) surpassed both the DECT parameters model (AUC = 0.741, *P* = 0.007) and the clinical-CT radiological features model (AUC = 0.880, *P* < 0.001). In a comparative analysis of the two spectral analysis methods, volumetric spectral analysis exhibited superior diagnostic efficacy compared to conventional spectral analysis, as shown in Table 4.

**Table 4:** Comparison of diagnostic performance of different prediction models in discriminating Ki-67 and TTF-1 expression

Model	Volumetric spectral analysis				Conventional spectral analysis			
	AUC	Sensitivity (%)	Specificity (%)	Accuracy (%)	AUC	Sensitivity (%)	Specificity (%)	Accuracy (%)
**Ki-67**								
C DECT	0.741	75.5	67.6	70.1	0.719	47.2	88.2	69.0
C Clinic	0.880	67.9	100.0	77.0	0.880	67.9	100.0	77.0
C Total	0.943	79.2	100.0	85.1	0.929	81.1	91.2	83.9
**TTF-1**								
C DECT	0.800	53.6	95.5	69.0	0.756	62.3	81.8	68.1
C Clinic	0.924	88.4	81.8	85.0	0.924	88.4	81.8	85.0
C Total	0.967	91.3	90.9	89.4	0.941	91.3	84.1	85.8

C _DECT_, DECT parameters model; C _Clinic_, clinical-CT radiological features model; C _Total_, the combined predictive model; AUC, the area under the curve

### Multivariate logistic analysis and prediction of TTF-1 expression status performance

The characteristics of the significant DECT parameters and radiological features between the different expression status of TTF-1 are summarized in Table 5 and S8 (See Supplementary Material for more details). As shown in Table S9, it revealed that CT_100keV_-VP (OR = e^8.556^, 95% CI: 5.571-e^15.394^, *P* = 0.014), CT_120keV_-VP (OR = 1/e^11.179^, 95% CI: 1/e^1.842^- 0.159, *P* = 0.019), Zeff-AP (OR = e^160.368^, 95% CI: e^44.456^- e^276.280^, *P* = 0.007), K_40 − 70_-AP (OR = 1/e^78.672^, 95% CI: e^29.268^- e^128. 076^, *P* = 0.002), AEF (OR = e^36.060^, 95% CI: 39.350- e^68.447^, *P* = 0.029), age (OR = 0.799, 95% CI: 0.678–0.941, *P* = 0.007), and histological type (OR = 1/e^6.808^, 95% CI: 1/e^3.492^-0.030, *P* < 0.001) were independent predictors of the TTF-1 predictive model. The detailed equations were as follows: logit (P) = 8.556 × CT_100keV_-VP + (-11.179) × CT_120keV_-VP + 160.368 × Zeff-AP + (-78.672) × K_40 − 70_-AP + 36.060 × AEF + (-0.224) × age + (-6.808) × histological type (ADC = 1; SQCC = 2)-4011.475, where ‘P’ is the probability of TTF-1 expression status. (the calculation formula of ‘P’ is shown in Supplemental Materials).

**Table 5:** Comparison of the spectral CT analysis results for TTF-1 expression

parameter	Volumetric spectral analysis			Conventional spectral analysis		
	TTF-1-negative (*n* = 44)	TTF-1-positive (*n* = 69)	*P* value	TTF-1-negative (*n* = 44)	TTF-1-positive (*n* = 69)	*P* value
**Arterial Phase**						
CT40-keV (HU)	122.92 ± 33.06	131.13 ± 28.58	0.099^*^	120.24 ± 33.03	133.67 ± 31.01	**0.031**
CT60-keV (HU)	68.56 ± 14.01	69.53 ± 12.93	0.709	67.77 ± 14.60	71.83 ± 13.65	0.137
CT70-keV (HU)	55.70 ± 9.93	54.93 ± 9.96	0.690	55.38 ± 10.87	57.19 ± 10.32	0.375
CT80-keV (HU)	47.47 ± 7.73	45.60 ± 8.60	0.246	47.43 ± 8.98	47.84 ± 8.82	0.813
CT100-keV (HU)	38.22 ± 6.14	35.12 ± 7.82	**0.012** ^*^	38.50 ± 7.73	37.32 ± 8.05	0.441
CT120-keV (HU)	33.78 ± 5.94	30.07 ± 7.84	**0.002** ^*^	34.21 ± 7.60	32.24 ± 8.14	0.200
CT140-keV (HU)	31.07 ± 6.01	27.02 ± 7.95	**0.001** ^*^	31.59 ± 7.68	29.18 ± 8.30	0.124
K40–70	2.24 ± 0.82	2.54 ± 0.71	**0.027** ^*^	2.16 ± 0.81	2.55 ± 0.78	**0.013**
K40–100	1.41 ± 0.52	1.60 ± 0.45	**0.026** ^*^	1.36 ± 0.51	1.61 ± 0.49	**0.013**
Zeff	8.30 ± 0.25	8.40 ± 0.22	**0.026** ^*^	8.30 ± 0.25	8.42 ± 0.24	**0.011**
IC (mg/cm^3^)	11.55 ± 4.36	13.18 ± 3.77	**0.027** ^*^	11.48 ± 4.30	13.55 ± 4.15	**0.012**
NIC	0.12 ± 0.04	0.15 ± 0.05	**0.001** ^*^	0.12 ± 0.04	0.16 ± 0.06	**0.001**
WC (mg/cm^3^)	1023.61 ± 6.63	1019.26 ± 8.64	**0.003** ^*^	1024.91 ± 7.83	1022.00 ± 9.13	0.116^*^
**Venous Phase**						
CT40-keV (HU)	157.84 ± 33.81	155.95 ± 37.78	0.788	154.86 ± 35.84	157.04 ± 37.48	0.760
CT60-keV (HU)	84.33 ± 13.50	81.63 ± 17.58	0.388	84.20 ± 15.19	82.90 ± 17.30	0.684
CT70-keV (HU)	67.24 ± 9.74	64.01 ± 13.23	0.140	67.25 ± 11.08	65.30 ± 13.12	0.415
CT80-keV (HU)	56.28 ± 7.99	52.75 ± 10.76	**0.048**	56.42 ± 9.02	54.17 ± 10.76	0.251
CT100-keV (HU)	43.97 ± 6.99	40.11 ± 8.60	**0.003** ^*^	44.29 ± 7.65	41.49 ± 9.03	0.051^*^
CT120-keV (HU)	38.02 ± 7.02	33.99 ± 7.93	**0.001** ^*^	38.41 ± 7.56	35.39 ± 8.58	0.059
CT140-keV (HU)	33.97 ± 8.13	30.34 ± 7.70	**0.001** ^*^	34.90 ± 7.68	31.75 ± 8.48	**0.048**
K40–70	3.02 ± 0.88	3.06 ± 0.87	0.792	2.92 ± 0.90	3.06 ± 0.88	0.425
K40–100	1.90 ± 0.54	1.93 ± 0.55	0.755	1.84 ± 0.56	1.93 ± 0.56	0.443
Zeff	8.53 ± 0.23	8.55 ± 0.26	0.592	8.54 ± 0.26	8.57 ± 0.26	0.488
IC (mg/cm^3^)	15.48 ± 4.18	15.97 ± 4.62	0.567	15.56 ± 4.72	16.26 ± 4.70	0.443
NIC	0.37 ± 0.08	0.42 ± 0.13	**0.011**	0.37 ± 0.10	0.43 ± 0.14	**0.013**
WC (mg/cm^3^)	1024.77 ± 7.94	1021.25 ± 7.77	**0.002** ^*^	1025.98 ± 8.23	1023.43 ± 8.58	0.121
ECV	0.23 ± 0.06	0.25 ± 0.08	0.079^*^	0.23 ± 0.07	0.26 ± 0.08	0.083
AEF	0.75 ± 0.21	0.85 ± 0.16	**0.008**	0.75 ± 0.22	0.84 ± 0.16	**0.003** ^*^
NAEF	0.33 ± 0.09	0.37 ± 0.11	**0.035** ^*^	0.33 ± 0.08	0.37 ± 0.12	0.070^*^

Data are presented as mean ± standard deviation; *, Mann-Whitney test; K_40**–**70_ = (CT_40 − keV_- CT_70 − keV_)/30; K_40**–**100_ = (CT_40 − keV_- CT_100 − keV_)/60; Zeff, effective atomic number; (N)IC, (normalized) iodine concentration; WC, water concentration; ECV(%)=(1-hematocrit)×NIC; (N)AEF, (normalized) arterial enhancement fraction

**Fig. 4:**
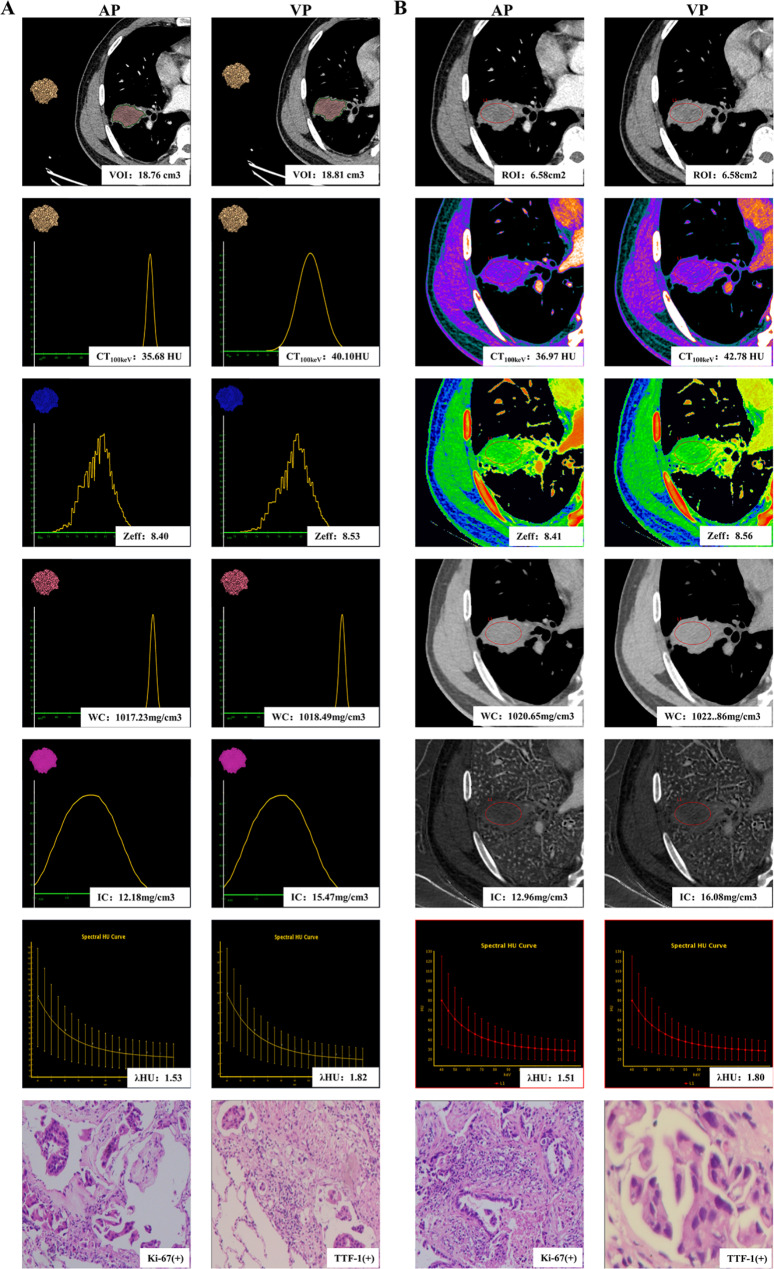
DECT images in a 54-year-old man with adenocarcinoma in the right lower lobe (Ki-67, high expression; TTF-1, positive). In the volumetric spectral analysis **(A)**, the whole lesion was completely outlined in the mediastinal window, with a volume of 18.76 cm^3^ in the arterial phase and a volume of 18.81 cm^3^ in the venous phase. In the conventional spectral analysis **(B)**, the circle indicates the region of interest outlining the lesion, with an ROI of 6.58cm^2^; VOI, the volume of interest; ROI, region of interest; AP, arterial phase; VP, venous phase

**Fig. 5:**
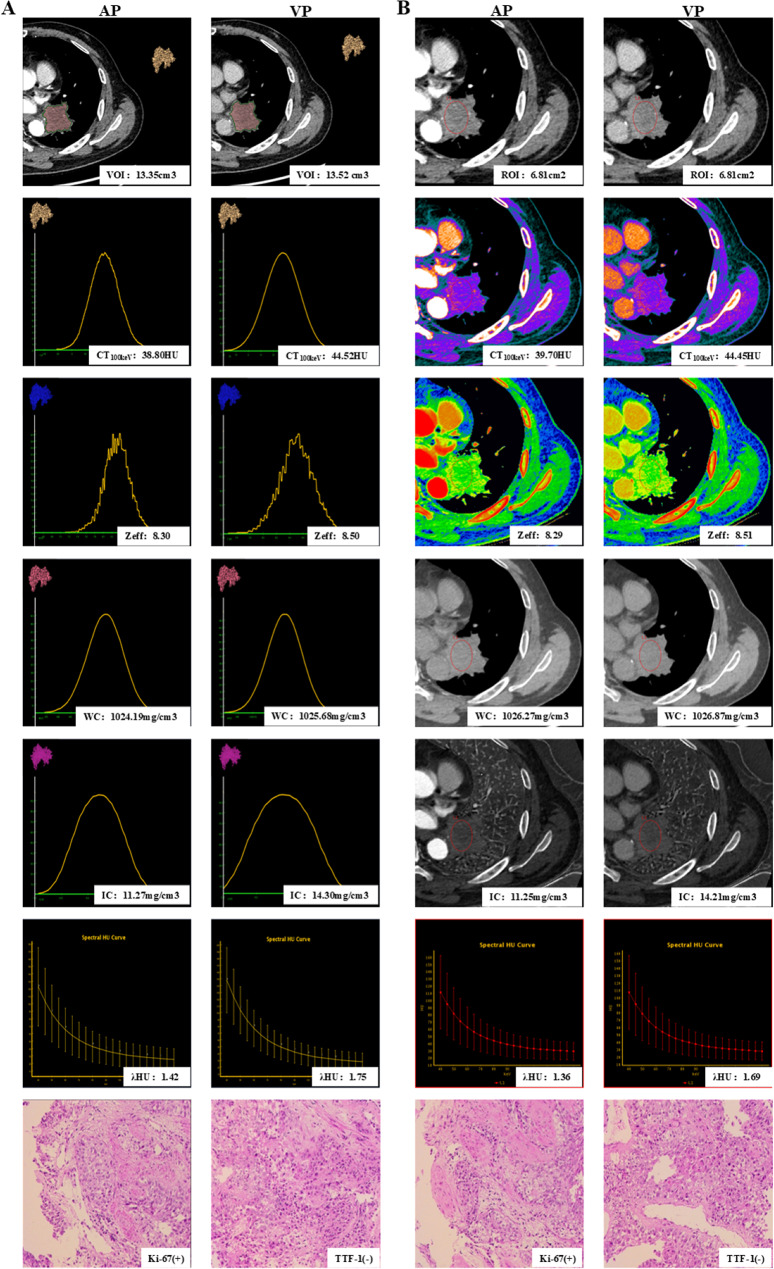
DECT images in a 63-year-old man with squamous cell carcinoma in the left lower lobe (Ki-67, high expression; TTF-1, negative). In the volumetric spectral analysis **(A)**, the whole lesion was completely outlined in the mediastinal window, with a volume of 13.35 cm^3^ in the arterial phase and a volume of 13.52 cm^3^ in the venous phase. In the conventional spectral analysis **(B)**, the circle indicates the region of interest outlining the lesion, with a ROI of 6.81cm^2^; VOI, the volume of interest; ROI, region of interest; AP, arterial phase; VP, venous phase

The Hosmer-Lemeshow goodness-of-fit test yielded a non-significant result (*P* = 0.689), suggesting an adequate fit of the model. The combined predictive model integrating all seven independent factors showed high diagnostic efficacy, with an AUC of 0.967, sensitivity of 91.3%, specificity of 90.9% and accuracy of 89.4%. As shown in Fig. 3B, the diagnostic performance of the combined prediction model (AUC = 0.967, *P* < 0.001) surpassed both the DECT parameters model (AUC = 0.800, *P* = 0.007) and the clinical-CT radiological features model (AUC = 0.924, *P* < 0.001). The diagnostic performance of volumetric spectral analysis was superior to that of conventional spectral analysis (Table 4). Representative cases are shown in Figs. 4 and 5.

## Discussion

With the rapid development of precision medicine, the choice of NSCLC treatment is highly dependent on pathological types and molecular phenotypes. The present study showed that the diagnostic performance of the combined prediction model, incorporating DECT parameters and clinical-CT radiological features, was useful in discriminating the expression of Ki-67 and TTF-1 in NSCLC, with AUC of 0.943 and 0.967, respectively. These quantitative parameters derived from spectral volumetric analysis can aid in accurate tumor differential diagnosis, thereby facilitating personalized treatment decision-making.

In terms of discriminating ADC from SQCC, our spectral analyses revealed that the IC of ADC were significantly higher than those of SQCC(13.69 mg/ml vs. 11.36 mg/ml), which is consistent with previous findings [28]. The IC value serves as a direct indicator of iodine-related information on perfusion and vascular distribution within tissues. ADC exhibits looser cancer tissues characterized by high micro-vessel density and abundant blood supply, resulting in a higher iodine concentration compared to SQCC [29]. Furthermore, our findings revealed that AEF exhibits superior diagnostic performance compared to other spectral parameters, which is consistent with the findings of Gao et al. [30]. This may be due to the fact that increased angiogenesis is positively correlated with increased contrast agent distribution in both the intravascular and extravascular compartments.

Compared with the volumetric spectral analysis, the diagnostic efficacy of the conventional spectral analysis was superior for the differential diagnosis of ADC and SQCC. One possible explanation is that ADC exhibits a greater abundance of mesenchymal components and small vessels compared to SQCC [28]. The iodine concentration in conventional spectral analysis remains unaffected by tumor necrosis, whereas three-dimensional whole-lesion analysis may introduce averaging effects in necrotic regions [31]. Conversely, volumetric analysis demonstrated superior diagnostic efficacy in discriminating Ki-67 and TTF-1 expression, potentially due to the fact that the two-dimensional region of interest may not reflect the tumor heterogeneity comprehensively [32].

Among molecular markers, Ki-67 is correlated with tumor proliferation, increased tumor size and progression. We found that Zeff was higher in the low Ki-67 expression group compared to the high expression group, in both arterial and venous phases. This aligns with previous findings reported by Mao et al. in gastric cancer [33]. Zeff reflects the total atomic number of a material mixture and exhibits a close relationship with the fundamental properties of the constituent element. Consistent with a study by Wen et al. [34], we found that the ECV-VP was lower in the high-level group than in the low-level group for Ki-67 expression. This may be due to the elevated proliferative activity in the high-level group, ultimately leading to poor cellular organization. Other CT features, notably lymph node enlargement, emerged as an independent predictor of Ki-67 expression in this work. Likewise, another study identified CT-reported local lymph node status as the most important radiological feature for differentiating the level of Ki-67 expression [34].

Previous studies have established TTF-1 expression is a good prognostic indicator for both OS and PFS in patients with advanced lung cancer [13, 35]. However, the role of TTF-1 in the pathogenesis and biology of lung cancer is uncertain. Among the DECT parameters, AEF, an indicator of tumor blood flow, emerged as a positive predictor of TTF-1 expression. This is likely attributed to the neovascularization in NSCLC, leading to an elevation in tumor microvascular density. We also found that the Zeff was statistically significant between TTF-1-positive and -negative groups (8.30 vs. 8.40). Furthermore, our findings suggest that the internal tissue conformation of the TTF-1 positive and negative groups was differently altered, in terms of tumor material components, density and cellular metabolic activity.

Our study has several limitations. First, this is a prospective study with a relatively small sample size from two institutions, which probably results in a selection bias. Second, this work focused on the relationship between DECT parameters and Ki-67 or TTF-1 expression, and further studies are needed to evaluate other immunohistochemical biomarkers, such as PD-L1 and NapsinA. Finally, our investigation was obtained with DECT device from a single vendor, future studies are warranted to validate the applicability of the DECT parameters from various manufacturers.

## Conclusions

In conclusion, our combined predictive model, which integrated DECT parameters using volumetric spectral analysis, demonstrated satisfactory diagnostic performance in differentiating Ki-67 and TTF-1 expression in patients with NSCLC, thereby providing valuable information for personalized treatment.

## Electronic supplementary material

Below is the link to the electronic supplementary material.

Supplementary Material 1

## Data Availability

No datasets were generated or analysed during the current study.

## Abbreviations

DECT: Dual-energy CT
NSCLC: Non-small cell lung cancer
ADC: Adenocarcinoma
SQCC: Squamous cell carcinoma
TTF-1: Thyroid transcription factor 1
ECV: Extracellular volume fraction
IC: Iodine concentration
NIC: Normalized iodine concentration
WC: Water concentration
Zeff: Effective atomic number
AEF: Arterial enhancement fraction
NAEF: Normalized arterial enhancement fraction
VOI: Volume of interest
ROI: Region of interest
AP: Arterial phase
VP: Venous phase
ICCs: Intraclass correlation coefficients
κ: Kappa index
ORs: Odds ratios
AUC: Area under the curve
95% CI: 95% Confidence interval
