# Supplementary material for: Differentiation of pathological subtypes and Ki-67 and TTF-1 expression by dual-energy CT (DECT) volumetric quantitative analysis in non-small cell lung cancer
Source: Cancer Imaging. 2024 Oct 25;24:146. doi: 10.1186/s40644-024-00793-6 (PMC11515807; doi:10.1186/s40644-024-00793-6)
Supplement: Supplementary file 1 — Supplementary Material 1 [file 40644_2024_793_MOESM1_ESM.docx]

**Supplementary Material**

**Comparison of DECT parameters between ADC and SQCC**

The results of volumetric spectral analysis showed that K_40-70_, K_40-100_, Zeff, and IC in AP and NIC in AP and VP of ADC were significantly higher than those of SQCC, whereas the CT_100keV_, CT_120keV_, CT_140keV_, and WC in AP and VP were significantly lower than those of SQCC (all P<0.05, **Table 2** and **Figure 2**,**3**). Similar to the results of volumetric spectral analysis, the conventional spectral analysis showed that the K_40-70_, K_40-100_, Zeff, and IC in AP and NIC in AP and VP of ADC were significantly higher than those in SQCC, while the CT_120keV_, CT_140keV_ and WC in AP and VP were significantly lower in ADC (all P<0.05). In addition, AEF and ECV in VP were also statistically different between the two groups (both P<0.05).

**Comparison of features for different expression levels of Ki-67**

The characteristics of DECT parameters and significant radiological features between groups with different levels of Ki-67 expression are summarized in **Tables 3** and **S6**. The results of volumetric spectral analysis showed that the K_40-70_, K_40-100_, Zeff, IC in AP and VP and CT_40keV_, NIC, ECV in VP were significantly higher in the low-level group compared to the high-level group (all P<0.05). Additionally, the CT_120keV_, CT_140keV_ and WC in AP were significantly higher in the high-level group. In contrast to the results of volumetric spectral analysis, conventional spectral analysis showed a statistically significant difference between the two groups in CT_40keV_ and NIC in AP (both P<0.05), whereas no significant difference was found in CT_120keV_ and CT_140keV_ in AP (P=0.155 and 0.112, respectively).

Among the clinical features evaluated, there were significant differences between the two groups in terms of gender, histological type, and smoking history (all P<0.05). Regarding the CT radiological features, there were significant differences between the two groups for pleural retraction and lymph node enlargement (both P<0.05), while no significant differences were found in other features.

**Comparison of features for different expression status of TTF-1**

The characteristics of the significant DECT parameters and radiological features between the different expression status of TTF-1 are summarized in **Table 4 and S7**. The results of volumetric spectral analysis showed that the CT_100keV_, CT_120keV_, CT_140keV_, WC in AP and VP and CT_80keV_ in VP were significantly higher in the TTF-1 negative group compared to the TTF-1 positive group. And the K_40-70_, K_40-100_, Zeff, IC, NIC in AP and NIC, AEF, NAEF in VP were significantly higher in the positive group. Unlike the results of volumetric spectral analysis, conventional spectral analysis showed significant differences between the two groups in CT_40keV_, K_40-70_, K_40-100_, Zeff, IC and NIC in AP and CT_140keV_, NIC and AEF in VP (all P<0.05), while the remaining DECT parameters were not statistically significant.

Among the clinical characteristics assessed, there were significant differences between the two groups with regard to age, gender, histological type, and smoking history (all P<0.05). In terms of CT radiological characteristics, significant differences between the two groups were found for tumor size and spiculation (both P<0.05), while no significant differences were found for other characteristics.

**Table S1** Clinical and CT morphological features analyzed in this study

| **Features** | **Definition** | **Scoring and definition** |
| --- | --- | --- |
| **Clinical features** |  |  |
| Age | Quantitative indicator | year |
| Gender | Male or Female | 1, male; 2, female |
| Histological type | Adenocarcinoma or Squamous cell carcinoma | 1, ADC; 2, SQCC |
| Smoking history | Smoker or Non-smoker | 0, absence; 1, presence |
| Drinking history | Drinker or Non-drinker | 0, absence; 1, presence |
| Family tumor history  **CT morphological features**  Tumor size  Lobulation  Spiculation  Air bronchogram  Pleural retraction  Obstructive change  Vascular invasion  Rim enhancement  Necrosis  Lymph node enlargement | Incidence of tumors in family  Mean of long and short diameters of the largest dimension on the lung window  A wavy or scalloped configuration of tumor’s surface  Lines radiating from the margins of the tumor  Tubelike or branched air structure within the tumor  Retraction of the pleura toward the tumor  Consolidation shadow caused by obstructive pneumonia or atelectasis at the edge of tumor  cancer cells penetrate and infiltrate the walls of blood vessels  A peripheral ring of increased density surrounding a central area of decreased density within a lesion  A hypo-attenuated central area on the non-enhanced images without enhancement during the postcontrast phases  Mediastinal or hilar lymph node enlargement，and maximum long-axis diameter ＞10mm | 0, absence; 1, presence  cm  0, absence; 1, presence  0, absence; 1, presence  0, absence; 1, presence  0, absence; 1, presence  0, absence; 1, presence  0, absence; 1, presence  0, absence; 1, presence  0, absence; 1, presence  0, absence; 1, presence |

**Table S2** Inter-observer reproducibility for tumor size and DECT parameters measurement

| **Parameter (Volumetric spectral analysis)** | **ICC (95% CI)** |
| --- | --- |
| **Tumor size**  **Arterial phase**  **Monochromatic CT numbers** | 0.957(0.925-0.975) |
| CT_40-keV_  CT_60-keV_ | 0.986(0.976-0.992)  0.983(0.970-0.990) |
| CT_70-keV_  CT_80-keV_  CT_100-keV_ | 0.984(0.972-0.991)  0.981(0.967-0.989)  0.983(0.970-0.990) |
| CT_120-keV_ | 0.962(0.934-0.978) |
| CT_140-keV_ | 0.986(0.976-0.992) |
| **Spectral curve slopes** |  |
| K_40_**_–_**_70_ | 0.987(0.978-0.993) |
| K_40_**_–_**_100_  **Zeff**  **IC**  **NIC**  **WC**  **Venous phase**  **Monochromatic CT numbers**  CT_40-keV_  CT_60-keV_  CT_70-keV_  CT_80-keV_  CT_100-keV_  CT_120-keV_  CT_140-keV_  **Spectral curve slopes**  K_40_**_–_**_70_  K_40_**_–_**_100_  **Zeff**  **IC**  **NIC**  **WC**  **ECV**  **AEF**  **NAEF** | 0.988(0.980-0.993)  0.989(0.981-0.994)  0.989(0.980-0.993)  0.888(0.810-0.935)  0.988(0.980-0.993)  0.979(0.963-0.988)  0.974(0.955-0.985)  0.972(0.952-0.984)  0.970(0.949-0.983)  0.972(0.951-0.984)  0.976(0.958-0.986)  0.978(0.962-0.988)  0.981(0.967-0.989)  0.984(0.971-0.991)  0.984(0.972-0.991)  0.984(0.971-0.991)  0.975(0.956-0.986)  0.984(0.972-0.991)  0.975(0.956-0.986)  0.976(0.958-0.986)  0.879(0.796-0.930) |

Note—Data are expressed as intraclass correlation coefficient with 95% CIs in parentheses.

DECT, Dual-energy CT; ICC, intraclass correlation coefficient; K_40_**_–_**_70_ = (CT_40-keV_- CT_70-keV_)/30; K_40_**_–_**_100_ = (CT_40-keV_- CT_100-keV_)/60; Zeff, effective atomic number; (N)IC, (normalized) iodine concentration; WC, water concentration; ECV(%)=(1-hematocrit)×NIC;(N)AEF, (normalized) arterial enhancement fraction.

**Table S3** Inter-observer reproducibility for tumor size and DECT parameters measurement

| **Parameter (Conventional spectral analysis)** | **ICC (95% CI)** |
| --- | --- |
| **Arterial phase**  **Monochromatic CT numbers** |  |
| CT_40-keV_  CT_60-keV_ | 0.987(0.977-0.993)  0.980(0.965-0.989) |
| CT_70-keV_  CT_80-keV_  CT_100-keV_ | 0.976(0.958-0.986)  0.970(0.947-0.983)  0.971(0.950-0.984) |
| CT_120-keV_ | 0.974(0.954-0.985) |
| CT_140-keV_ | 0.976(0.958-0.986) |
| **Spectral curve slopes** |  |
| K_40_**_–_**_70_ | 0.991(0.984-0.995) |
| K_40_**_–_**_100_  **Zeff**  **IC**  **NIC**  **WC**  **Venous phase**  **Monochromatic CT numbers**  CT_40-keV_  CT_60-keV_  CT_70-keV_  CT_80-keV_  CT_100-keV_  CT_120-keV_  CT_140-keV_  **Spectral curve slopes**  K_40_**_–_**_70_  K_40_**_–_**_100_  **Zeff**  **IC**  **NIC**  **WC**  **ECV**  **AEF**  **NAEF** | 0.991(0.985-0.995)  0.991(0.983-0.995)  0.992(0.985-0.995)  0.785(0.650-0.872)  0.987(0.978-0.993)  0.986(0.976-0.992)  0.985(0.973-0.991)  0.984(0.971-0.991)  0.979(0.963-0.988)  0.981(0.966-0.989)  0.981(0.967-0.989)  0.982(0.968-0.990)  0.987(0.978-0.993)  0.987(0.978-0.993)  0.988(0.978-0.993)  0.983(0.970-0.990)  0.971(0.950-0.984)  0.987(0.977-0.993)  0.974(0.954-0.985)  0.946(0.907-0.969)  0.851(0.752-0.913) |

Note—Data are expressed as intraclass correlation coefficient with 95% CIs in parentheses.

DECT, Dual-energy CT; ICC, intraclass correlation coefficient; AP, arterial phase; VP, venous phase; λ HU, slope rate of spectral curve; K_40_**_–_**_70_ = (CT_40-keV_- CT_70-keV_)/30; K_40_**_–_**_100_ = (CT_40-keV_- CT_100-keV_)/60; Zeff, effective atomic number; (N)IC, (normalized) iodine concentration; WC, water concentration; ECV(%)=(1-hematocrit)×NIC; (N)AEF, (normalized) arterial enhancement fraction.

**Table S4** Inter-observer agreement of CT radiological features

| **Subjective CT morphological features** | **Kappa** |
| --- | --- |
| Lobulation | 1.000 |
| Spiculation | 0.752 |
| Air bronchogram | 0.767 |
| Pleural retraction | 0.874 |
| Obstructive change | 0.819 |
| Vascular invasion | 0.750 |
| Rim enhancement | 0.778 |
| Necrosis | 0.934 |
| Lymph node enlargement | 0.848 |

**Table S5** Comparison of diagnostic performance for significant DECT parameters in histological types

| Significant Parameters | Volumetric spectral analysis | | | |  | Conventional spectral analysis | | | |
| --- | --- | --- | --- | --- | --- | --- | --- | --- | --- |
|  | AUC | Sensitivity (%) | Specificity (%) | Cut-off |  | AUC | Sensitivity (%) | Specificity (%) | Cut-off |
| **AP**  CT_100-keV_  CT_120-keV_  CT_140-keV_  K_40–70_  K_40–100_  Zeff  IC  NIC  WC  **VP**  CT_100-keV_  CT_120-keV_  CT_140-keV_  NIC  WC  ECV  AEF  **C_DECT_** | 0.649  0.683  0.703  0.645  0.645  0.644  0.643  0.653  0.695  0.665  0.691  0.703  0.641  0.680  0.616  0.666  0.709 | 50.0  66.7  58.3  63.1  63.1  63.1  63.1  61.9  61.9  54.8  61.9  71.4  50.0  70.2  54.8  61.9  72.6 | 80.0  68.6  77.1  71.4  71.4  71.4  71.4  68.6  71.4  80.0  74.3  68.6  85.7  65.7  77.1  71.4  62.9 | 35.1  33.4  29.3  2.4  1.5  8.3  12.2  0.1  1022.5  40.6  36.9  35.3  0.4  1025.5  0.2  0.8  - |  | -  0.632  0.645  0.662  0.662  0.664  0.663  0.677  0.670  -  0.613  0.615  0.653  0.596  0.618  0.677  0.801 | -  60.7  70.2  69.0  69.0  67.9  71.4  70.2  63.1  -  64.3  53.6  46.4  53.6  53.6  84.5  75.0 | -  62.9  54.3  65.7  65.7  65.7  65.7  65.7  68.6  -  62.9  71.4  82.9  68.6  71.4  60.0  77.1 | -  34.3  33.3  2.2  1.4  8.3  11.6  0.1  1025.4  -  38.4  32.9  0.4  1025.5  0.3  0.7  - |

Data are presented as the values; AP, arterial phase; VP, venous phase; K_40_**_–_**_70_ = (CT_40-keV_- CT_70-keV_)/30; K_40_**_–_**_100_ = (CT_40-keV_- CT_100-keV_)/60; Zeff, effective atomic number; (N)IC, (normalized) iodine concentration; WC, water concentration; ECV(%)=(1-hematocrit)×NIC; AEF, arterial enhancement fraction. AUC, the area under the curve; C_DECT_, Combined DECT Parameters Model.

**Table S6** Comparison of significant clinical and CT radiological features for Ki-67 expression

| Significant Features | Low-level | High-level | P value |
| --- | --- | --- | --- |
| **Clinical features ^a^** |  |  |  |
| Gender |  |  | 0.001**^b^** |
| Male  Female | 18(52.94)  16(47.06) | 45(84.91)  8(15.09) |  |
| Histological types |  |  | ＜0.001 **^b^** |
| Adenocarcinoma  Squamous cell carcinoma | 34(100.00)  0(0.00) | 33(62.26)  20(37.74) |  |
| Smoking history |  |  | 0.006 **^b^** |
| Yes | 13(38.24) | 36(67.92) |  |
| No | 21(61.76) | 17(32.08) |  |
| **CT radiological features ^a^**  Pleural retraction  Presence  Absence  Lymph node enlargement  Presence  Absence | 26(76.47)  8(23.53)  23(67.65)  11(32.35) | 18(33.96)  35(66.04)  46(86.79)  7(13.21) | 0.004 **^b^**  0.031 **^b^** |

**^a^** Categorical variable, data in parentheses are percentage.

**^b^** Chi-square test or Fisher’s exact probability method.

**Table S7** Multivariate analysis of variables associated with Ki-67 expression

| Variables | OR | 95% CI | P value |
| --- | --- | --- | --- |
| Zeff-AP | e^43.710^ | 3.473- e^86.175^ | 0.044 |
| Zeff-VP | 1/e^180.668^ | 1/e^339.530^-1/e^221.806^ | 0.026 |
| IC-VP | e^9.424^ | 2.345- e^17.995^ | 0.031 |
| ECV-VP  Gender  Pleural retraction  Lymph node enlargement | e^52.531^  0.123  0.079  11.626 | 31.784- e^101.604^  0.018-0.820  0.014-0.433  1.150-117.538 | 0.036  0.030  0.003  0.038 |

OR, odds ratio; CI, confidence interval

**Table S8** Comparison of significant clinical and CT radiological features for TTF-1 expression

| Significant Features | TTF-1-negative | TTF-1-positive | P value |
| --- | --- | --- | --- |
| **Clinical features** |  |  |  |
| Age (years) **^a^** | 66.95±8.57 | 60.57±9.05 | ＜0.001 **^d^** |
| Gender **^b^** |  |  | 0.001 **^c^** |
| Male  Female | 42(95.45)  2(4.54) | 48(69.57)  21(30.43) |  |
| Histological types **^b^** |  |  | ＜0.001 **^c^** |
| Adenocarcinoma  Squamous cell carcinoma | 13(29.55)  31(70.45) | 66(95.65)  3(4.34) |  |
| Smoking history **^b^** |  |  | 0.022 **^c^** |
| Yes | 33(75.00) | 37(53.62) |  |
| No | 11(25.00) | 32(46.38) |  |
| **CT radiological features**  Tumor size **^a^**  Spiculation **^b^**  Presence  Absence | 4.94±1.73  30(68.18)  14(31.82) | 3.68±1.27  60(86.96)  9(13.04) | ＜0.001 **^d^**  0.016 **^c^** |

**^a^** Data are presented as the mean ± standard deviation.

**^b^** Categorical variable, data in parentheses are percentage.

**^c^** Chi-square test or Fisher’s exact probability method.

**^d^** Two independent sample t-test

**Table S9** Multivariate analysis of variables associated with TTF-1 expression

| Variables | OR | 95% CI | P value |
| --- | --- | --- | --- |
| CT_100-keV_-VP | e^8.556^ | 5.571-e^15.394^ | 0.014 |
| CT_120-keV_-VP | 1/e^11.179^ | 1/e^1.842^- 0.159 | 0.019 |
| Zeff-AP  K_40–70_-AP | e^160.368^  1/e^78.672^ | e^44.456^- e^276.280^  e^29.268^- e^128. 076^ | 0.007  0.002 |
| AEF  age  Histological type | e^36.060^  0.799  1/e^6.808^ | 39.350- e^68.447^  0.678-0.941  1/e^3.492^-0.030 | 0.029  0.007  ＜0.001 |

OR, odds ratio; CI, confidence interval

**Table S10** DeLong test of AUC for different prediction models

| Model comparison | Volumetric spectral analysis |  | Conventional spectral analysis |
| --- | --- | --- | --- |
|  | P value |  | P value |
| **Ki-67**  C_DECT_ vs. C_Clinic_  C_DECT_ vs. C_Total_  C_Clinic_ vs. C_Total_  **TTF-1**  C_DECT_ vs. C_Clinic_  C_DECT_ vs. C_Total_  C_Clinic_ vs. C_Total_ | 0.020  ＜0.001  0.019  0.004  ＜0.001  0.044 |  | 0.004  ＜0.001  0.067  0.001  ＜0.001  0.243 |

C_DECT_, DECT parameters model; C_Clinic_, clinical-CT radiological features model; C_Total_, the combined predictive model; AUC, the area under the curve.

**The calculation formula of the total combined predictive model for predicting the probability (*P*) of Ki-67 expression level**

*P* = exp(t)/[exp(t) + 1]

t = 43.710 × Zeff-AP + (-180.668) × Zeff-VP + 9.424 × IC-VP + 52.531 × ECV-VP + (-2.099) × gender (male=1; female=2) + (-2. 540) × pleural retraction (presence=1; absence=0) + 2.453× lymph node enlargement (presence=1; absence=0)-1725.321

**The calculation formula of the total combined predictive model for predicting the probability (*P*) of TTF-1 expression status**

*P* = exp(t)/[exp(t) + 1]

t =8.556 × CT_100keV_-VP + (-11.179) × CT_120keV_-VP + 160.368 × Zeff-AP + (-78.672) × K_40-70_-AP + 36.060 × AEF + (-0.224) × age + (-6.808) × histological type (ADC=1; SQCC=2)-4011.475
